# Comparing the Efficacy of Two Cognitive Screening Tools in Identifying Gray and White Matter Brain Damage among Older Adults

**DOI:** 10.1155/2024/5527225

**Published:** 2024-04-23

**Authors:** Paula Andreatta Maduro, Manoel Pereira Guimarães, Mateus de Sousa Rodrigues, Ana Paula Pereira Rolim Coimbra Pinto, Américo Alves da Mota Junior, Alaine Souza Lima Rocha, Juliana Magalhães Duarte Matoso, Bruno Bavaresco Gambassi, Paulo Adriano Schwingel

**Affiliations:** ^1^Post-Graduation Program in Health Sciences (PPGCS), University of Pernambuco (UPE), Recife, PE 50100-130, Brazil; ^2^Human Performance Research Laboratory (LAPEDH), UPE, Petrolina, PE 56328-900, Brazil; ^3^University Hospital of the Federal University of Vale do São Francisco (HU-UNIVASF), Brazilian Hospital Services Company (EBSERH), Petrolina, PE 56304-205, Brazil; ^4^Department of Physical Therapy, Federal University of Ceará (UFC), Fortaleza, CE 60430-450, Brazil; ^5^Department of Clinical Medicine, Pedro Ernesto University Hospital, State University of Rio de Janeiro (UERJ), Rio de Janeiro, RJ 20551-030, Brazil; ^6^Post-Graduation Program in Management of Health Programs and Services (PPGGPSS), CEUMA University (UNICEUMA), São Luís, MA 65075-120, Brazil

## Abstract

**Background:**

Ageing is associated with structural changes in brain regions and functional decline in cognitive domains. Noninvasive tools for identifying structural damage in the brains of older adults are relevant for early treatment.

**Aims:**

This study aims to evaluate and compare the accuracy of the Mini-Mental State Examination (MMSE) and Montreal Cognitive Assessment (MoCA©) in identifying gray and white matter brain damage in older individuals with varying degrees of cognitive impairment.

**Methods:**

Ninety older adults (62 women) with an average age of 69 ± 7 years were enrolled and categorized as having no cognitive impairment (NCI), mild cognitive impairment (MCI), or moderate cognitive impairment (MoCI). Magnetic resonance imaging (MRI) was utilized to assess the number, volume, and distribution of brain damage. The Fazekas and Scheltens scales were applied to the brain MRIs, and inferential statistics were employed to compare variables among the groups.

**Results:**

Cognitive impairment was observed in 56.7% of the participants (95% confidence interval (CI): 46.4–66.4%), with thirty-six older adults (40%) classified as MCI and 15 (17%) as MoCI. Cognitive impairment and medial temporal lobe (MTL) atrophy were found to be associated (*p*=0.001), exhibiting higher mean volume scales of the MTL atrophied area in the MoCI group (*p* < 0.001). The MMSE accurately revealed MTL atrophy based on the Scheltens (*p* < 0.05) and Fazekas (*p* < 0.05) scales. At the same time, the MoCA accurately identified periventricular white matter (PWM) abnormalities according to the Fazekas scale (*p* < 0.05).

**Conclusions:**

The MMSE and MoCA screening tools effectively identified gray and white matter brain damage in older adults with varying degrees of cognitive impairment. Lower MMSE scores are associated with MTL atrophy and lesions, and lower MoCA scores are related to PWM lesions. The concurrent use of MMSE and MoCA is recommended for assessing structural changes in distinct brain regions.

## 1. Introduction

Brain atrophy arises from a complex interplay of genetic, environmental, and lifestyle factors, leading to structural and functional decline in both white and gray matter [1–3]. White matter atrophy, characterised by the degradation of myelinated axons and oligodendrocytes, disrupts neural connectivity and information transmission [4]. Risk factors associated with white matter atrophy encompass cardiovascular risk factors, genetic predisposition, inflammatory processes, and lifestyle factors [3]. Conversely, protective strategies such as physical exercise, cognitive stimulation, and dietary interventions have been correlated with reduced white matter atrophy and the preservation of cognitive function Gray matter atrophy predominantly affects neuronal cell bodies and dendrites, resulting in synaptic loss and neuronal degeneration [5]. Risk factors linked to gray matter atrophy include ageing, neurodegenerative diseases, exposure to environmental toxins, and psychological stress [3]. Protective mechanisms against gray matter atrophy involve cognitive engagement, social interaction, and mindfulness-based interventions, which facilitate neuroplasticity and enhance resilience against neurodegenerative processes [1, 2].

It is estimated that over half of the brain's tissue volume comprises gray and white matter fibers [6]. The assessment of gray and white matter is preferably conducted through magnetic resonance imaging (MRI) [5, 7], given that this technique serves as the gold standard for detecting neuronal structural changes, particularly those associated with small vessel diseases [8]. The evaluation of damage to gray and white matter is optimally achieved using T2-weighted MRI with fluid-attenuated inversion recovery (FLAIR) sequences [7, 9], which stands as the method of choice for analysing white matter [10]. Recent research has indicated that FLAIR sequences can effectively characterise subtle microstructural features of neural tissue [11].

During the late 1980s, correlations were discovered between white and gray matter changes and declining cognitive functions [9]. Both gray matter atrophy [12] and diminished white matter integrity [13, 14] have been linked to cognitive impairment, particularly among older adults. Although white matter lesions are present in both young and older individuals with normal cognitive function, their progression is closely associated with dementia and represents an independent risk factor for the development of dementia syndrome [15–17]. Moreover, prior studies have demonstrated that white matter lesions can give rise to subtle cognitive deficits in older individuals without cognitive impairment [15, 18].

In a clinical context, the Mini-Mental State Examination (MMSE) [19] and the Montreal Cognitive Assessment (MoCA©) [20] serve as instruments for screening cognitive impairment. The MMSE, developed by Folstein et al. [19], remains one of the most widely used screening tools for cognitive impairment [21, 22]. It consists of various tasks assessing orientation, registration, attention, calculation, recall, and language [19, 23]. Despite its popularity, the MMSE has notable limitations [24, 25]. First, its brevity restricts the assessment to a few cognitive domains, overlooking nuanced deficits in executive function, visuospatial abilities, and higher-order reasoning. Second, the MMSE is susceptible to educational and cultural biases, as performance may be influenced by literacy levels and language proficiency. Furthermore, the ceiling effect of the MMSE renders it less sensitive to mild cognitive impairment [26], leading to false-negative results, particularly in highly educated individuals [25]. These limitations underscore the need for supplementary assessments to capture a comprehensive profile of cognitive function.

The MoCA, developed by Nasreddine et al. [20], was designed to address some of the shortcomings of the MMSE [19]. It includes tasks assessing visuospatial abilities, executive function, attention, language, memory, and orientation, offering a more comprehensive evaluation of cognitive function [20, 27]. In addition, the MoCA incorporates tasks sensitive to mild cognitive impairment, enhancing its diagnostic accuracy in detecting subtle cognitive deficits [28]. However, the MoCA is not without limitations. Its administration time is longer compared to the MMSE, potentially limiting its feasibility in busy clinical settings. Moreover, although the MoCA reduces educational bias compared to the MMSE [29], cultural and linguistic factors may still influence performance, particularly in diverse populations.

In this context, MoCA [20] has exhibited sensitivity in predicting future cognitive decline [27] and adapted and validated versions existing for different populations, including a Brazilian Portuguese variant [29]. The MMSE [19], also possesses a version adapted for the Brazilian population [30], with a cut-off point adjusted according to the individual's level of education, which can act as a confounding variable in MMSE results [19, 30]. Pinto et al. [29] suggested that educational level should also be taken into consideration when evaluating MoCA results.

While the MMSE demonstrates good sensitivity, particularly in older age groups [21, 22], cognitive changes identified by MoCA are associated with white matter lesions [31]. Despite the MMSE being considered a measure of global cognition [19, 23], the MoCA offers the advantage of better observation of executive function tasks [20]. However, few studies have investigated the relationship between these two cognitive impairment screening tools and structural damage in the gray and white matter of older adults as assessed by MRI. According to Li et al. [32], the results of both MMSE and MoCA can be indicative of brain imaging diagnosis. Yet, these authors asserted that MoCA exhibits greater sensitivity for detecting structural damage in white and gray matter. In contrast, Wang et al. [33] found no significant correlation between MMSE results and white matter volume in the four brain regions assessed by MRI. The authors reported only weak statistical correlations between MoCA scores and white matter volume in those same regions [33]. The enhanced sensitivity of MoCA, when compared to MMSE, likely stems from its ability to evaluate a wider range of cognitive domains and items of greater complexity [28]. Considering the considerations, the objective of this study was to assess and compare the precision of MMSE and MoCA© in identifying gray and white matter brain damage among older adults with varying degrees of cognitive impairment.

## 2. Materials and Methods

### 2.1. Study Design

This observational, cross-sectional, and analytical study was conducted using the translated [34] and updated [35] version of Strengthening the Reporting of Observational Studies in Epidemiology (STROBE) [36] and the STROBE Statement Guidelines for reporting observational studies [37].

The study followed the ethical principles of the Declaration of Helsinki (1964) and Resolution 466/2012 of the Brazilian National Health Council. The study received approval from the Research Ethics Committee under Certificate of Presentation for Ethical Consideration (CAAE) no. 38942320.4.0000.5192, issued on November 10, 2020.

### 2.2. Study Location

The study was carried out in a municipality in the interior of the northeast of Brazil, in the Sertão region. The tests and exams used in the study were carried out at the Hospital Universitário da Universidade Federal do Vale do São Francisco (HU-Univasf), which is part of the Empresa Brasileira de Serviços Hospitalares (EBSERH). Ambulatory patients were recruited at the Polyclinic of the HU-Univasf, between January and September 2022, and data collection occurred during the same period.

### 2.3. Participants

The study included people aged 60 years or over, which is the definition of elderly adults in developing countries according to the World Health Organization [38], of both sexes, regardless of marital status, with four or more years of schooling, regardless of family income.

The following parameters were adopted as exclusion criteria: (a) elderly patients with a sum of >18 points on the Beck Depression Inventory (BDI) [39]; (b) elderly patients with uncorrected motor or sensory deficits that made it impossible to perform neuropsychological tests; (c) patients submitted to a recent change in therapeutic regimen, within the past four weeks; (d) patients using psychotropic medication; (e) elderly patients on four or more antihypertensive drugs; (f) elderly patients with systolic blood pressure ≥180 mmHg and/or diastolic blood pressure ≥110 mmHg; (g) patients with a history of angina, acute myocardial infarction, invasive cardiovascular procedures, heart transplants, or presence of pacemakers; (h) elderly patients diagnosed with Parkinson's disease; (i) patients with a history of stroke or transient ischemic attack; and (j) elderly patients with untreated hypothyroidism.

The Clinical Dementia Rating (CDR) scale, initially developed by Hughes et al. [40] and subsequently refined by Morris [41], served as the assessment tool utilized by the psychiatrist from HU-Univasf for patient classification. Participants in the study were categorized into three groups based on their level of cognitive impairment, as determined by the psychiatrist: (a) no cognitive impairment (NCI); (b) mild cognitive impairment (MCI); and (c) moderate cognitive impairment (MoCI). Patients were classified as NCI if they had a CDR score of 0. Conversely, those with a CDR score of 2 were classified as MoCI.

### 2.4. Variables Analysed

Socioeconomic data and general health status were evaluated. Anthropometric measurements were taken. Resting blood pressure was recorded, and MMSE and MoCA were applied. Clinical and functional evaluations were performed by using the Katz et al. [42] and Lawton scales [43], and executive function and information processing speed were evaluated by using the trail-making test, parts A and B. Finally, an MRI was also performed.

A team of trained professionals blindly tabulated and duplicated all information about the analysed variables. Qualitative variables were coded, and quantitative variables were tabulated up to two decimal places according to the International System of Units.

### 2.5. Assessments

The socioeconomic evaluation was conducted using a structured questionnaire based on the criteria established by the Brazilian Institute of Geography and Statistics (IBGE). This questionnaire included inquiries about sociodemographic factors such as age, marital status, self-reported race/skin colour, occupation, and education, as well as socioeconomic information such as monthly family income in terms of minimum wages.

The general health status was measured with the help of a structured questionnaire having questions referring to the patient's personal history, general health conditions, and medication use. The anthropometric evaluation consisted of measuring total body mass and height using, respectively, a portable HBF-214 digital scale (Omron Healthcare Inc., Lake Forest, IL, United States of America (USA)) with a precision of 0.1 kg and a maximum weight capacity of 150 kg and a portable scientific stadiometer (Sanny, São Bernardo do Campo, SP, Brazil) with a precision of 0.1 cm and a maximum length of 2.20 meters. Using these data, the body mass index (BMI) was evaluated using the formula as follows: total body mass (kg)/height (m)^2^.

Blood pressure was measured using an automatic HEM-711 equipment (Omron Healthcare Inc.), which consists of an electronic digital arm blood pressure measurement device, with automatic inflation and deflation. The measurement method of this device is oscillometric, ranging from 0 to 280 mmHg. For this measurement, patients remained in the sitting position for ten minutes, following the guidelines of the current Brazilian Guidelines on Arterial Hypertension [44]. Three consecutive measurements were taken, with one-minute intervals on the nondominant arm, with adequate cuff size for arm circumference. The average of the three measurements was used.

The assessment of physical activity levels was conducted using the International Physical Activity Questionnaire (IPAQ), a tool that has been validated in a representative sample of the Brazilian population [45]. For estimating participants' physical activity levels, this study employed the short version of the questionnaire, which comprises inquiries about the frequency and duration of physical activity, including walking and engagement in activities of moderate and vigorous intensity. The IPAQ was utilized to categorize participants' physical activity levels as either “active,” “insufficiently active,” or “sedentary.”

In the assessment of executive function and information processing speed, we utilized the trail-making test (TMT), encompassing parts A and B. TMT part A was employed to gauge information processing speed, while TMT part B was administered to evaluate executive function. Each part consists of twenty-five circles distributed across a sheet of paper. In part A, the circles are sequentially numbered from 1 to 25, and participants are required to draw lines connecting the numbers in an ascending order. In part B, the circles feature both numbers (1 to 13) and letters (A to L). Like part A, participants are instructed to draw lines connecting the circles in an ascending pattern, where they must link the first number to the first letter, and so on (1-A-2-B-3-C). Participants are directed to complete the connections as swiftly as possible, without lifting the pen or pencil from the paper. If a mistake is made, the evaluator promptly identifies it and permits the subject to rectify it. The time taken for error correction is included in the overall completion time for the task. The test concludes either when the participant completes the sequence or opts to discontinue the test [46].

The clinical evaluation and functional assessment were conducted by utilizing the Katz et al. [42] and Lawton [43] scales. The Katz scale appraises the level of independence in executing fundamental activities of daily living (ADLs). This scale encompasses six items that gauge an individual's proficiency in self-care activities, organized according to a hierarchy of complexity: feeding, continence, transfer, personal hygiene, dressing, and bathing [42]. On the other hand, the Lawton scale comprises eight items intended to assess an individual's capability to perform instrumental activities of daily living (IADLs), which encompasses using the telephone, shopping, food preparation, housekeeping, laundry, transportation, managing medications, and financial handling. The reduced scores for each item indicate a greater individual reliance. The scoring ranges from 7 to 21 points, with higher scores indicating a better performance [43]. Within this study, participants with scores of ≤11 were classified as dependent in terms of IADLs.

### 2.6. Cognitive Impairment

The MMSE [19] and MoCA© [20] were employed for assessing cognitive impairment. Compared to alternative assessment instruments, these tests offer a harmonious blend of sensitivity, specificity, ease of administration, and comprehensive evaluation, making them the preferred options for discerning cognitive impairment within clinical and research contexts [21, 22, 27]. While alternative tests may exhibit specific advantages in selecting cognitive domains or demographic groups, the widespread adoption and rigorous validation of the MMSE and MoCA have firmly established them as the foremost tools for cognitive assessment.

The MMSE is a widely used cognitive screening test that assesses various cognitive domains such as orientation, memory, recall, attention, naming objects, following verbal and written commands, writing a sentence, and copying a figure [19, 30]. For the MMSE, the following severity levels of cognitive impairments were adopted as cut-off points [47–49]: NCI (MMSE between 24 and 30 points); MCI (MMSE between 20 and 23 points); and MoCI (MMSE between 13 and 19 points). In addition, the study adjusted the NCI cut-off criteria based on years of education as follows [30, 49]: for participants with 1–4 years of education, the cut-off was 25 points; for those with 5–8 years, 26.5 points; for those with 9–11 years, 28 points; and those with 12 or more years, 29 points.

Despite being a more recent development, MoCA scores also range from 0 to 30 points. This neuropsychological test includes more complex tasks, such as executive function, and assesses orientation, drawing figures, processing speed, naming objects, memory, recall, attention, vigilance, repetition, verbal fluency, and abstraction [20, 27]. In the validation study, a cut-off score of 26 or more points was established to detect cognitive impairment [20]. For the MoCA, the following severity levels of cognitive impairments were adopted as cut-off points [50]: NCI (MoCA between 27 and 30 points); MCI (MoCA between 21 and 26 points); and MoCI (MoCA between 18 and 20 points). In this neuropsychological test, one point is added for individuals with 12 or fewer years of education [29, 50]. Similarly to MMSE, MoCA cut-offs were adjusted based on the years of education, using the values proposed by Pinto et al. [29], who evaluated this instrument in the Brazilian population.

### 2.7. Magnetic Resonance Imaging (MRI)

All subjects underwent MRI to evaluate brain volume scales in a clinic specializing in diagnostic imaging following a standard protocol. Volumetric (T1), FLAIR, susceptibility-weighted imaging (SWI), standard axial T2 diffusion-weighted imaging (DWI), and T2 acquisitions (titled axial and coronal plane for the hippocampus) were performed in a SIGNA™ Explorer 1.5T (GE Healthcare, Wauwatosa, WI, USA).

The Fazekas scale [51] was used to analyse white matter lesions and to evaluate the amount of chronic ischemic changes of small vessels in the older persons enrolled in the study. The scale divides the white matter into periventricular white matter (PWM) and deep white matter (DWM), and each region receives a grade depending on the size and confluence of the lesions. PWM was evaluated as follows: 0 = absent; 1 = pencil-thin lining; 2 = smooth halo; and 3 = irregular periventricular signal extending into the DWM. DWM was evaluated as follows: 0 = absent; 1 = punctate; 2 = beginning confluence; and 3 = large confluent areas [51]. For visual analysis of parietal atrophy, the Koedam score [52] was used, generating a scale from 0 to 3, as follows: grade 0: closed sulcus, without gyral atrophy; grade 1: mild sulcal widening and mild gyral atrophy; grade 2: substantial sulcal enlargement and substantial gyral atrophy; and grade 3: marked sulcal widening and knife blade gyral atrophy [52]. To obtain the visual classification of medial temporal lobe atrophy on coronal T1-weighted MRI, the Scheltens score was used [53], with scores ranging from 0 to 4, where 0 = no cerebrospinal fluid is visible around the hippocampus; 1 = choroid fissure is slightly widened; 2 = moderate widening of the choroid fissure, mild enlargement of the temporal horn, and mild loss of hippocampal height; 3 = marked widening of the choroid fissure, moderate enlargement of the temporal horn, and moderate loss of hippocampal height; and 4 = marked widening of the choroid fissure, marked enlargement of the temporal horn, and the hippocampus is markedly atrophied and internal structure is lost [53].

### 2.8. Statistical Methods

The sample size for this study was calculated with the results obtained in the pilot study for the volumetry of the medial temporal lobe on MRI by using the following formula: *n*=2*σ*^2^[*Zα*+*Zβ*]^2^ ÷ *δ*^2^. The pooled standard deviation (*ϭ*) was 0.777 cm^3^; *α* was 5% (*Zα* = 1.96); *β* was 20% (*Zβ* = 0.84), and the mean difference (*δ*) was 0.107 cm^3^. According to this formula, the estimated sample size (*n*) totals eighty-eight participants.

The obtained data were entered into the Statistical Package for the Social Sciences for Windows (SPSS) computer program (SPSS Inc., Chicago, IL, USA, release 16.0.2, 2008) using a double-entry method, with checks conducted for consistency and adherence to the specified range [54]. Descriptive statistical analysis was employed, representing categorical variables as absolute and relative frequencies and continuous variables as means ± standard deviations after confirming data normality through the Kolmogorov–Smirnov test. The occurrence of cognitive impairment was expressed as a percentage, and the precision was quantified by a 95% confidence interval (95% CI). Comparative analysis between participants with and without cognitive impairment encompassed numerical variables, utilizing a one-way analysis of variance (ANOVA) followed by Tukey's post hoc test. For categorical variables, Pearson's chi-square test and Fisher's exact test were applied. All statistical analyses were conducted as two-tailed tests, and statistical significance was determined at a level of *p* ≤ 0.05.

## 3. Results

The study encompassed 90 older adults, of whom 62 were women, with a mean age of 69.0 ± 6.5 years and a mean BMI of 28.1 ± 5.0 kg/m^2^. The prevalence of cognitive impairment within the sample was estimated to be 56.7% (95% CI: 46.4%–66.4%). Among the participants, 39 (28 women) were categorized as having NCI, 36 (27 women) as exhibiting MCI, and 15 (7 women) as having MoCI. The prevalence of MCI and MoCI in the sample was 40.0% (95% CI: 30.5%–50.3%) and 16.7% (95% CI: 10.3%–25.8%), respectively.

Older adults with MoCI displayed a significantly higher mean age than those with NCI and MCI (*p* < 0.001). Furthermore, participants with NCI exhibited a higher mean number of school years than the other groups (*p*=0.007). Statistically significant differences in means were observed among the groups (*p* < 0.001) for both ADLs and IADLs results. Superior outcomes were evident in older adults with NCI, followed by those with MCI (Table 1). Statistically significant disparities (*p* < 0.05) between the three groups were also observed in executive function (TMT part B) and cognitive impairment, as identified by the MMSE and MoCA screening tools. Older adults with NCI exhibited statistically superior scores compared to those with MCI, and MCI patients had higher scores than those with MoCI (*p* < 0.05). Patients with NCI achieved a higher mean score (*p* < 0.05) in information processing speed (TMT part A). No statistical differences were detected among the three groups for other clinical and demographic variables.

Upon analysing the MRI results, a statistically significant association (*p*=0.001) emerged between cognitive impairment and medial temporal lobe atrophy (Table 2). MRI results of older adults with NCI corresponded to the absence of visible cerebrospinal fluid around the hippocampus, indicative of normal conditions. In contrast, MRI results of cognitive impairment patients correlated with indicators of structural damage, such as choroid fissure enlargement, temporal horn enlargement, and loss of hippocampal height (Scheltens scale).

The brain MRI volumetric quantification revealed that older adults with MoCI exhibited greater mean volume scales of atrophied areas in the medial temporal lobe when compared to the other groups (Table 3).

When applying the MMSE to screen all participants for cognitive impairment, fifty-three older adults (40 women) were classified as NCI, 31 (19 women) as MCI, and 6 (3 women) as MoCI. The MMSE yielded different cognitive impairment frequencies within the sample compared to the psychiatrist's reference values. Patients with MCI and MoCI demonstrated higher medial temporal lobe atrophy volume scale scores than older adults with NCI screened by the MMSE (Table 4).

Applying the MoCA to screen for cognitive impairment, thirty-two older adults (23 women) were classified as NCI, 13 (10 women) as MCI, and 45 (29 women) as MoCI. Regarding the proportion of participants classified according to the reference values adopted by the psychiatrist in the assessment of cognitive impairment, the MoCA overestimated the number of participants with MoCI. In addition, brain MRI volumetric quantification identified that patients with MoCI screened by the MoCA had a higher mean of medial temporal lobe atrophy volume scale than those with NCI (Table 5).

A consistent statistical association (*p*=0.04) persisted between cognitive impairment and medial temporal lobe atrophy (Table 6). Those classified as NCI by the MMSE had MRI results aligned with the absence of visible cerebrospinal fluid around the hippocampus. In contrast, those with cognitive impairment displayed more pronounced structural damage, as evidenced by higher scale scores.

A statistical association (*p*=0.01) was found between cognitive impairment (MCI and MoCI) and the presence of neuronal structural damage in the PWM (Fazekas scale), demonstrating that participants with NCI had a higher frequency of tests within the normal range in comparison to the other groups (Table 7).

## 4. Discussion

The key findings of this study are twofold. First, the cognitive impairment screening tools MMSE and MoCA demonstrated accuracy in predicting neuronal structural damage in the medial temporal lobe (Scheltens and Fazekas scales) and periventricular white matter (Fazekas scale) of older adults' brains. Lower MMSE scores are associated with the presence of lesions in the medial temporal lobe (Fazekas scale) and a higher volume of atrophy, as indicated by brain MRI's volumetric quantification (Scheltens scale). Simultaneously, lesions in the periventricular white matter (Fazekas scale) are associated with lower MoCA scores. Second, the frequencies of cognitive impairment classified using the cut-off points of the MMSE and MoCA screening tools differed from each other and the reference values set by the psychiatrist.

Although the MMSE and MoCA are among the most popular and widely utilized screening instruments for cognitive decline within the systematic evaluation of geriatric patients in healthcare centres, these examinations often exhibit inaccuracies and unreliability, stemming from either human error or patients' physical limitations in correctly interpreting questions alongside motor deficits [49]. Our findings indicate that while the MMSE tended to overestimate the number of participants with NCI, the MoCA tended to overestimate the number of participants with cognitive impairments. Notably, since motor deficits were considered exclusion criteria, the misclassification observed with these cognitive screening tools seems to be associated with educational and cultural biases [29, 55–57]. Moreover, it is noteworthy to mention the MMSE's limited sensitivity in detecting mild or moderate cognitive impairment, particularly in the early stages of neurodegenerative diseases such as Alzheimer's disease [55, 56]. Similarly, the MoCA employs more stringent scoring criteria compared to the MMSE, often featuring lower cut-off scores for impairment in some test versions [20, 27, 29, 57, 58]. This disparity in scoring criteria may contribute to a higher proportion of individuals being categorized as having cognitive impairment when assessed with the MoCA [59, 60].

While the total scores of MMSE and MoCA diverged in classifying cognitive impairments, both tools still detected better structural outcomes in the brains of older adults with NCI and worse outcomes in those with MoCI. In this regard, this study aligns with the literature, which recognizes both tests as having good accuracy in assessing cognitive impairment [23, 29, 61]. Conversely, cross-analysis results suggest that these tools should not be employed in isolation, given that MMSE identified a greater proportion of participants with NCI, while MoCA overestimated the presence of MoCI. MMSE exhibited low sensitivity in screening for MCI or MoCI within the sample, while MoCA displayed low accuracy in screening participants with MCI. These findings concur with prior research indicating MoCA's better ability to detect cognitive heterogeneity within the sample [22, 61, 62].

When participants were categorized using the psychiatrist's reference values, the MoCI group showed a higher prevalence of moderate and marked choroid fissure widening. Conversely, when older adults were screened using MMSE cut-off points, the test's ability to differentiate the lowest score on the Scheltens scale for medial temporal atrophy was evident. Similarly, classification using the MoCA revealed a statistically significant difference in terms of medial temporal atrophy between the NCI and MoCI groups. These findings are in line with the recent studies [29]. According to Roh et al. [63], medial temporal atrophy could be an early indicator of cognitive decline, while Zhang et al. [64] suggested that such atrophy might contribute to impairments in semantic learning strategies.

The medial temporal lobe plays a vital role in memory formation, emotional regulation, and spatial navigation [65–67]. Atrophy in this region can lead to memory impairments, potentially progressing to conditions such as Alzheimer's disease [65, 66]. In addition, changes in emotional regulation linked to medial temporal lobe atrophy may contribute to mood disturbances, such as depression and anxiety, common in older adults [68]. On the other hand, the white matter acts as the brain's communication network, facilitating signal transmission between regions [69, 70]. PWM abnormalities, often associated with conditions such as vascular dementia and small vessel disease, can lead to executive dysfunction, memory deficits, and mood changes [71, 72]. These abnormalities may worsen mental health conditions and increase the risk of stroke and other cerebrovascular events in older adults [18, 69–72], highlighting the importance of addressing them for preserving cognitive function and mental well-being.

In addition, white matter is a pertinent parameter in assessing cognitive impairment [6, 9]. Regarding periventricular white matter assessment via MRI, irregular periventricular signals extending to the deep white matter (Fazekas grade 3) were observed in the MoCI group. Participants with MCI exhibited pencil-thin lines on periventricular white matter (Fazekas grade 1), and those with NCI displayed no alterations (Fazekas grade 0). These results affirm the link between white matter changes and cognitive decline [13, 14].

Considering the associations unveiled in this study, a dual approach involving both MMSE and MoCA is proposed to screen for the risk of cognitive decline swiftly and accurately. This is due to both tools showing links with medial temporal atrophy (Scheltens scale) and periventricular white matter lesions (Fazekas scale) observed in MRI scans. This approach holds promise for early diagnosis of neuronal structural damage, benefiting older adults, their families, and society. Li et al. [32] emphasized in their study that results from both MMSE and MoCA are associated with neuronal structural damage assessed through imaging examinations. Conversely, Wang et al. [33] found weak statistical correlations (*r* ≤ 0.40) between MoCA values and white matter volume across four brain regions assessed by MRI.

There is a noteworthy difference in the systematic review by Pinto et al. [57], who, despite the varying accuracy between studies, identified that more than 80% of the papers showed that MoCA is superior to MMSE in detecting patients with MCI and NCI. Moreover, compared with MMSE, the same authors recommended MoCA as the preferred test for cognitive screening in elderly patients, particularly those with MCI. The prevalence of cognitive decline as screened by both tools underscores the importance of considering educational level-adjusted MoCA cut-off points within the study population, aligning with Pinto et al. [57]. Conversely, the application of education-based cut-off points for MMSE [30] may have led to more elderly patients being classified as NCI, a situation that warrants further investigation.

Regarding the sample, this study identified a lower prevalence of individuals in the MCI group, with this group tending to be older. These outcomes coincide with the epidemiology of natural ageing and the gradual decline in cognitive function [73]. Notably, the severity of cognitive decline diminishes the survival rate of elderly populations [74], particularly when linked to comorbidities [75]. In addition, older individuals tend to progress to more advanced disease stages [76]. Furthermore, this investigation reveals that less severe conditions might be linked to higher education levels, which is considered a protective factor against cognitive decline, reinforcing the findings of prior research [77–79].

Another significant consideration is the role of ADLs and IADLs as pivotal factors in the assessment of cognitive impairment, particularly in low- and middle-income countries, thus complementing the evaluation [80]. This study demonstrated a progressive decline in ADLs and IADLs scores as cognitive impairment stages advanced, alongside varying outcomes observed in parts A and B of the TMT. Depending on the severity of cognitive impairment, participants exhibited elevated scores on both test parts, indicating compromised processing speed and diminished executive function. Furthermore, all participants required more time to complete TMT part B, likely due to its heightened cognitive demands compared to part A [81, 82]. However, while the TMT provides valuable insights into the executive function, relying exclusively on it may overlook other critical facets of this multifaceted construct [83]. This exclusive reliance on the TMT may yield an incomplete understanding of the executive function, potentially leading to the misinterpretation of cognitive profiles [84, 85]. Moreover, the TMT may lack sensitivity in detecting subtle deficits, particularly in individuals with mild cognitive impairment or early-stage neurodegenerative disorders [85]. In addition, nonexecutive factors such as motor speed, visual scanning abilities, and processing speed can influence TMT performance, potentially leading to the misinterpretation of cognitive performance due to these factors [81–85].

One strength of this study lies in its demonstration of the effectiveness of two noninvasive cognitive impairment screening tools in assessing structural damage in the white and gray matter among a relatively large sample of elderly patients. However, our study is not without limitations, beginning with its design. The groups were categorized solely by the degree of cognitive impairment and not by the affected cognitive domain (amnestic and nonamnestic) or neurocognitive disorder subtype (such as Alzheimer's disease, Lewy body disease, Parkinson's disease, vascular disease, frontotemporal lobar degeneration, and traumatic brain injury, among others). Another previously discussed limitation is the inaccuracies and limited reliability demonstrated by the MMSE and MoCA in detecting mild or moderate cognitive impairments. Furthermore, a potential confounding factor arises from the higher number of years of education in the NCI group. To mitigate this bias, the study adopted education-adjusted cut-off points for cognitive impairment in both the applied tools. Similarly, depression in the study sample was controlled for, as it could serve as a clinically significant behavioural or psychological alteration (comorbidity) for neurocognitive disorders. Finally, some agitated patients, particularly those with cognitive decline, might poorly tolerate MRI, leading to motion artefacts that compromise image quality and interpretation. Efforts to minimize this concern included using faster sequences with motion artifact correction, such as periodically rotated overlapping parallel lines with enhanced reconstruction (PROPELLER) sequence.

## 5. Conclusions and Implications

In conclusion, our study highlights the effectiveness of using both the MMSE and MoCA as screening tools for cognitive impairment in older adults. By demonstrating their ability to predict neuronal structural damage, particularly in the medial temporal lobe and PWM, observed through MRI scans, we emphasize the importance of a dual approach involving both tests for swift and accurate screening. This strategy not only facilitates early detection of cognitive decline but also plays a vital role in mitigating cognitive deterioration, thereby promoting the well-being of ageing individuals and benefiting their families and society.

Further investigation into the role of ADLs and IADLs in assessing cognitive impairment, as well as exploration of alternative screening tools, can enhance the understanding of cognitive function in older adults. Moving forward, future research should delve deeper into specific cognitive domains affected by cognitive impairment and various neurocognitive disorder subtypes. Longitudinal studies are needed to explore the progression of cognitive decline over time and its correlation with neuronal structural changes.

Overall, our study contributes valuable insights into both basic and applied research, reaffirming the importance of cognitive screening tools in clinical practice and research settings. By addressing limitations and building upon strengths identified in this study, future research can further advance our understanding of cognitive impairment and improve diagnostic accuracy, leading to enhanced quality of care for older adults worldwide.

## Figures and Tables

**Table 1:** Sociodemographic and clinical variables of older adults (*N* = 90) with no cognitive impairment (NCI), mild cognitive impairment (MCI), or moderate cognitive impairment (MoCI).

Variables	NCI	MCI	MoCI	*P* value
	(*n* = 39)	(*n* = 36)	(*n* = 15)	
Age (years)	66.2 ± 4.0^A^	69.8 ± 6.5^A^	74.1 ± 8.3^B^	<0.001^*∗*^
*Sex, n (%)*				
Male	11 (28.2)	9 (25.0)	8 (53.3)	0.120
Female	28 (71.8)	27 (75.0)	7 (46.7)	
Schooling (years)	9.6 ± 3.1^A^	7.4 ± 3.1^B^	7.5 ± 3.2^B^	0.007^*∗*^
*Monthly salary, n (%)*				
1 to 2 minimum wages	23 (59.0)	28 (77.8)	13 (86.7)	0.069
>2 minimum wages	16 (41.0)	8 (22.2)	2 (13.3)	
*Level of physical activity, n (%)*				
Sedentary	8 (20.5)	7 (19.4)	4 (26.7)	0.797
Insufficiently active	16 (41.0)	19 (52.8)	7 (46.7)	
Active	15 (28.5)	10 (27.8)	4 (26.7)	
Presence of chronic disease, *n* (%)	30 (76.9)	29 (80.6)	12 (80.0)	0.922
Coronavirus infection, *n* (%)	12 (30.8)	9 (25.0)	1 (6.7)	0.181
Tobacco use, *n* (%)	0 (0.0)	2 (5.6)	1 (6.7)	0.299
Alcohol use, *n* (%)	4 (10.3)	2 (5.6)	2 (13.3)	0.622
Total body mass (kg)	72.3 ± 16.3	68.7 ± 12.9	67.0 ± 14.2	0.396
Height (cm)	159.2 ± 6.6	155.5 ± 7.1	157.6 ± 8.3	0.084
Body mass index (kg/m^2^)	28.4 ± 5.2	28.4 ± 4.9	26.9 ± 4.5	0.572
Systolic blood pressure (mmHg)	115.9 ± 13.2	117.3 ± 10.1	112.9 ± 16.0	0.522
Diastolic blood pressure (mmHg)	74.1 ± 8.5	74.9 ± 7.2	71.9 ± 9.5	0.491
Baseline heart rate (bpm)	65.6 ± 11.4	65.1 ± 9.9	61.9 ± 13.4	0.539
Amount of medication (*n*)	2.9 ± 1.9	2.4 ± 1.8	3.3 ± 2.3	0.299
Activities of daily living (ADLs) (*n*)	6.0 ± 0.0^A^	4.8 ± 0.5^B^	3.1 ± 0.9^C^	<0.001^*∗*^
Instrumental ADLs (*n*)	25.8 ± 0.8^A^	23.7 ± 1.4^B^	17.6 ± 2.9^C^	<0.001^*∗*^
Beck Depression Inventory (*n*)	12.8 ± 8.0	13.0 ± 4.6	9.6 ± 5.1	0.194
Trail-making test part A	50.5 ± 19.1^A^	75.4 ± 42.5^A^	123.73 ± 66.4^B^	<0.001^*∗*^
Trail-making test part B	150.6 ± 65.1^A^	265.5 ± 110.6^B^	333.63 ± 113.7^C^	<0.001^*∗*^
Mini-Mental State Examination (*n*)	26.5 ± 2.4^A^	23.8 ± 2.7^B^	18.9 ± 4.4^C^	<0.001^*∗*^
Montreal Cognitive Assessment (*n*)	22.0 ± 2.8^A^	16.3 ± 2.6^B^	12.9 ± 4.3^C^	<0.001^*∗*^

^*∗*^Indicate statistically significant values. Means ± standard deviation (SD) with different letters represents statistically significant values (*p* < 0.05) in the comparison between groups according to the Tukey's post hoc test.

**Table 2:** Prevalence of structural changes on magnetic resonance imaging in older adults (*N* = 90) with no cognitive impairment (NCI), mild cognitive impairment (MCI), or moderate cognitive impairment (MoCI).

Brain tissue-specific and region-specific abnormalities	NCI	MCI	MoCI	*P* value
	(*n* = 39)	(*n* = 36)	(*n* = 15)	
*Periventricular white matter, n (%)*				
No lesions	11 (28.2)	6 (16.7)	2 (13.3)	0.220
Pencil-thin lining	16 (41.0)	19 (52.8)	8 (53.3)	
Smooth halo	11 (28.2)	6 (16.7)	2 (13.3)	
Irregular periventricular signal extending into the deep white matter	1 (2.6)	5 (13.9)	3 (20.0)	
*Deep white matter, n (%)*				
No lesions	11 (28.2)	8 (22.2)	3 (20.0)	0.625
Punctuate	16 (41.0)	18 (50.0)	7 (46.7)	
Beginning confluence	10 (25.6)	6 (16.7)	2 (13.3)	
Large confluent areas	2 (5.1)	4 (11.1)	3 (20.0)	
*Parietal atrophy, n (%)*				
Closed sulcus, without gyral atrophy	16 (41.0)	15 (41.7)	3 (20.0)	0.476
Mild sulcal widening and mild gyral atrophy	16 (41.0)	17 (47.2)	8 (53.3)	
Substantial sulcal enlargement and substantial gyral atrophy	7 (17.9)	4 (11.1)	4 (26.7)	
*Medial temporal lobe atrophy, n (%)*				
No cerebrospinal fluid is visible around the hippocampus	20 (51.3)	16 (44.4)	2 (13.3)	0.001^*∗*^
Choroid fissure is slightly widened	15 (38.5)	14 (38.9)	4 (26.7)	
Moderate widening of the choroid fissure, mild enlargement of the temporal horn, and mild loss of hippocampal height	3 (7.7)	6 (16.7)	5 (33.3)	
Marked widening of the choroid fissure, moderate enlargement of the temporal horn, and moderate loss of hippocampal height	1 (2.5)	0 (0.0)	4 (26.7)	
*Global cortical atrophy, n (%)*				
Normal volume and no ventricular enlargement	16 (41.0)	12 (33.3)	3 (20.0)	0.296
Opening of sulci and mild ventricular enlargement	13 (33.3)	17 (47.2)	8 (53.3)	
Volume loss of gyri and moderate ventricular enlargement	10 (25.6)	5 (13.9)	4 (26.7)	
“Knife blade” atrophy and severe ventricular enlargement	0 (0.0)	2 (5.6)	0 (0.0)	

^*∗*^Indicate statistically significant values (*p* < 0.05).

**Table 3:** Brain magnetic resonance imaging volumetric quantification of older adults (*N* = 90) with no cognitive impairment (NCI), mild cognitive impairment (MCI), or moderate cognitive impairment (MoCI).

Brain tissue-specific and region-specific abnormalities	NCI (*n* = 39)	MCI (*n* = 36)	MoCI (*n* = 15)	*P* value
	Mean ± SD	Mean ± SD	Mean ± SD	
Periventricular white matter (cm^3^)	1.05 ± 0.83	1.28 ± 0.91	1.40 ± 0.99	0.350
Deep white matter (cm^3^)	1.08 ± 0.87	1.17 ± 0.91	1.33 ± 1.05	0.653
Parietal atrophy (cm^3^)	0.77 ± 0.74	0.69 ± 0.67	1.07 ± 0.70	0.230
Medial temporal lobe atrophy (cm^3^)	0.62 ± 0.75^A^	0.72 ± 0.74^A^	1.73 ± 1.03^B^	<0.001^*∗*^
Global cortical atrophy (cm^3^)	0.85 ± 0.81	0.92 ± 0.84	1.07 ± 0.70	0.668

^*∗*^Indicate statistically significant values. Means ± standard deviation (SD) with different letters represents statistically significant values (*p* < 0.05) in the comparison between groups according to the Tukey's post hoc test.

**Table 4:** Brain magnetic resonance imaging volumetric quantification of older adults (*N* = 90) with no cognitive impairment (NCI), mild cognitive impairment (MCI), or moderate cognitive impairment (MoCI) also screened by the Mini-Mental State Examination (MMSE).

Brain tissue-specific and region-specific abnormalities	NCI (*n* = 53)	MCI (*n* = 31)	MoCI (*n* = 6)	*P* value
	Mean ± SD	Mean ± SD	Mean ± SD	
Periventricular white matter (cm^3^)	1.23 ± 0.82	1.13 ± 0.99	1.33 ± 1.03	0.831
Deep white matter (cm^3^)	1.21 ± 0.89	1.03 ± 0.95	1.33 ± 1.03	0.621
Parietal atrophy (cm^3^)	0.77 ± 0.70	0.74 ± 0.77	1.17 ± 0.41	0.400
Medial temporal lobe atrophy (cm^3^)	0.58 ± 0.75^A^	1.13 ± 0.96^B^	1.67 ± 0.82^B^	0.001^*∗*^
Global cortical atrophy (cm^3^)	0.91 ± 0.82	0.90 ± 0.83	1.00 ± 0.63	0.962

^*∗*^Indicate statistically significant values. Means ± standard deviation (SD) with different letters represents statistically significant values (*p* < 0.05) in the comparison between groups according to the Tukey's post hoc test.

**Table 5:** Brain magnetic resonance imaging volumetric quantification of older adults (*N* = 90) with no cognitive impairment (NCI), mild cognitive impairment (MCI), or moderate cognitive impairment (MoCI) also screened by the Montreal cognitive assessment (MoCA).

Brain tissue-specific and region-specific abnormalities	NCI (*n* = 32)	MCI (*n* = 13)	MoCI (*n* = 45)	
	Mean ± SD	Mean ± SD	Mean ± SD	
Periventricular white matter (cm^3^)	1.06 ± 0.80	1.08 ± 0.64	1.33 ± 1.00	0.367
Deep white matter (cm^3^)	1.09 ± 0.86	1.00 ± 0.71	1.24 ± 1.00	0.625
Parietal atrophy (cm^3^)	0.84 ± 0.72	0.46 ± 0.66	0.84 ± 0.71	0.201
Medial temporal lobe atrophy (cm^3^)	0.63 ± 0.80^A^	0.54 ± 0.52^A,B^	1.09 ± 0.97^B^	0.029^*∗*^
Global cortical atrophy (cm^3^)	0.87 ± 0.80	0.69 ± 0.75	1.00 ± 0.83	0.458

^*∗*^Indicate statistically significant values. Means ± standard deviation (SD) with different letters represents statistically significant values (*p* < 0.05) in the comparison between groups according to the Tukey's post hoc test.

**Table 6:** Prevalence of structural changes on magnetic resonance imaging in older adults (*N* = 90) with no cognitive impairment (NCI), mild cognitive impairment (MCI), or moderate cognitive impairment (MoCI) also screened by the Mini-Mental State Examination (MMSE).

Brain tissue-specific and region-specific abnormalities	NCI	MCI	MoCI	*P* value
	(*n* = 53)	(*n* = 31)	(*n* = 6)	
*Periventricular white matter, n (%)*				
No lesions	10 (18.9)	8 (25.8)	1 (16.7)	0.211
Pencil-thin lining	24 (45.3)	16 (51.6)	3 (50.0)	
Smooth halo	16 (30.2)	2 (6.5)	1 (16.7)	
Irregular periventricular signal extending into the deep white matter	3 (5.7)	5 (16.1)	1 (16.7)	
*Deep white matter, n (%)*				
No lesions	12 (22.6)	9 (29.0)	1 (16.7)	0.369
Punctuate	22 (41.5)	16 (51.6)	3 (50.0)	
Beginning confluence	15 (28.3)	2 (6.5)	1 (16.7)	
Large confluent areas	4 (7.5)	4 (12.9)	1 (16.7)	
*Parietal atrophy, n (%)*				
Closed sulcus, without gyral atrophy	20 (37.7)	14 (45.2)	0 (0.0)	0.237
Mild sulcal widening and mild gyral atrophy	25 (47.2)	11 (35.5)	5 (83.2)	
Substantial sulcal enlargement and substantial gyral atrophy	8 (15.1)	6 (19.4)	1 (16.7)	
*Medial temporal lobe atrophy, n (%)*				
No cerebrospinal fluid is visible around the hippocampus	29 (54.6)	9 (29.0)	0 (0.0)	0.039^*∗*^
Choroid fissure is slightly widened	18 (34.0)	12 (38.7)	3 (50.0)	
Moderate widening of the choroid fissure, mild enlargement of the temporal horn, and mild loss of hippocampal height	5 (9.4)	7 (22.6)	2 (33.3)	
Marked widening of the choroid fissure, moderate enlargement of the temporal horn, and moderate loss of hippocampal height	1 (1.9)	3 (9.7)	1 (16.7)	
*Global cortical atrophy, n (%)*				
Normal volume, no ventricular enlargement	19 (35.8)	11 (35.5)	1 (16.7)	0.921
Opening of sulci and mild ventricular enlargement	21 (39.6)	13 (41.9)	4 (66.7)	
Volume loss of gyri and moderate ventricular enlargement	12 (22.6)	6 (19.4)	1 (16.7)	
“Knife blade” atrophy and severe ventricular enlargement	1 (1.9)	1 (3.2)	0 (0.0)	

^*∗*^Indicate statistically significant values (*p* < 0.05).

**Table 7:** Prevalence of structural changes on magnetic resonance imaging in older adults (*N* = 90) with no cognitive impairment (NCI), mild cognitive impairment (MCI), or moderate cognitive impairment (MoCI) also screened by the Montreal Cognitive Assessment (MoCA).

Brain tissue-specific and region-specific abnormalities	NCI	MCI	MoCI	*P* value
	(*n* = 32)	(*n* = 13)	(*n* = 45)	
*Periventricular white matter, n (%)*				
No lesions	9 (28.1)	2 (15.4)	8 (17.8)	0.012^*∗*^
Pencil-thin lining	12 (37.5)	8 (61.5)	23 (51.1)	
Smooth halo	11 (34.4)	3 (23.1)	5 (11.1)	
Irregular periventricular signal extending into the deep white matter	0 (0.0)	0 (0.0)	9 (20.0)	
*Deep white matter, n (%)*				
No lesions	9 (28.1)	3 (23.1)	10 (22.2)	0.107
Punctuate	12 (37.5)	7 (53.8)	22 (48.9)	
Beginning confluence	10 (31.3)	3 (23.1)	5 (11.1)	
Large confluent areas	1 (3.1)	0 (0.0)	8 (17.8)	
*Parietal atrophy, n (%)*				
Closed sulcus, without gyral atrophy	11 (34.4)	8 (61.5)	15 (33.3)	0.438
Mild sulcal widening and mild gyral atrophy	15 (46.9)	4 (30.8)	22 (48.9)	
Substantial sulcal enlargement and substantial gyral atrophy	6 (18.8)	1 (7.7)	8 (17.8)	
*Medial temporal lobe atrophy, n (%)*				
No cerebrospinal fluid is visible around the hippocampus	17 (53.1)	6 (46.2)	15 (33.3)	0.122
Choroid fissure is slightly widened	11 (34.4)	7 (53.8)	15 (33.3)	
Moderate widening of the choroid fissure, mild enlargement of the temporal horn, and mild loss of hippocampal height	3 (9.4)	0 (0.0)	11 (24.4)	
Marked widening of the choroid fissure, moderate enlargement of the temporal horn, and moderate loss of hippocampal height	1 (3.1)	0 (0.0)	4 (8.9)	
*Global cortical atrophy, n (%)*				
Normal volume, no ventricular enlargement	12 (37.5)	6 (46.2)	13 (28.9)	0.692
Opening of sulci and mild ventricular enlargement	12 (37.5)	5 (38.5)	21 (46.7)	
Volume loss of gyri and moderate ventricular enlargement	8 (25.0)	2 (15.4)	9 (20.0)	
“Knife blade” atrophy and severe ventricular enlargement	0 (0.0)	0 (0.0)	2 (4.4)	

^*∗*^Indicate statistically significant values (*p* < 0.05).

## Data Availability

The data used to support the findings of this study are available from the corresponding author upon request.

## References

[1] Turrini S., Wong B., Eldaief M. (2023). The multifactorial nature of healthy brain ageing: brain changes, functional decline and protective factors. *Ageing Research Reviews*.

[2] Habes M., Janowitz D., Erus G. (2016). Advanced brain aging: relationship with epidemiologic and genetic risk factors, and overlap with Alzheimer disease atrophy patterns. *Translational Psychiatry*.

[3] Bishop N. A., Lu T., Yankner B. A. (2010). Neural mechanisms of ageing and cognitive decline. *Nature*.

[4] Zhou J., Zhang P., Zhang B., Kong Y. (2022). White matter damage in Alzheimer’s disease: contribution of oligodendrocytes. *Current Alzheimer Research*.

[5] Kassem M. S., Lagopoulos J., Stait-Gardner T. (2013). Stress-induced gray matter loss determined by MRI is primarily due to loss of dendrites and their synapses. *Molecular Neurobiology*.

[6] Sampaio-Baptista C., Johansen-Berg H. (2017). White matter plasticity in the adult brain. *Neuron*.

[7] Wang J., Chen Y., Liang H. (2019). The role of disturbed small-world networks in patients with white matter lesions and cognitive impairment revealed by resting state function Magnetic Resonance Images (rs-fMRI). *Medical Science Monitor*.

[8] Ferguson K. J., Cvoro V., MacLullich A. M. J. (2018). Visual rating scales of white matter hyperintensities and atrophy: comparison of computed tomography and magnetic resonance imaging. *Journal of Stroke and Cerebrovascular Diseases*.

[9] Steingart A., Hachinski V. C., Lau C. (1987). Cognitive and neurologic findings in subjects with diffuse white matter lucencies on computed tomographic scan (Leuko-Araiosis). *Archives of Neurology*.

[10] Badji A., Westman E. (2020). Cerebrovascular pathology in Alzheimer’s disease: hopes and gaps. *Psychiatry Research: Neuroimaging*.

[11] Bahsoun M. A., Khan M. U., Mitha S. (2022). FLAIR MRI biomarkers of the normal appearing brain matter are related to cognition. *NeuroImage: Clinica*.

[12] Villemagne V. L., Chételat G. (2016). Neuroimaging biomarkers in Alzheimer’s disease and other dementias. *Ageing Research Reviews*.

[13] Alber J., Alladi S., Bae H. (2019). White matter hyperintensities in vascular contributions to cognitive impairment and dementia (VCID): knowledge gaps and opportunities. *Alzheimer’s and Dementia: Translational Research & Clinical Interventions*.

[14] Ji F., Pasternak O., Liu S. (2017). Distinct white matter microstructural abnormalities and extracellular water increases relate to cognitive impairment in Alzheimer’s disease with and without cerebrovascular disease. *Alzheimer’s Research & Therapy*.

[15] Drumond L. D. S., Barbosa L. B. d. S., Passini M. C. (2020). A relação entre idosos hipertensos e déficit cognitivo. *Revista Eletrônica Acervo Saúde*.

[16] Kalaria R. N. (2016). Neuropathological diagnosis of vascular cognitive impairment and vascular dementia with implications for Alzheimer’s disease. *Acta Neuropathologica*.

[17] Schmidt R., Ropele S., Enzinger C. (2005). White matter lesion progression, brain atrophy, and cognitive decline: the Austrian stroke prevention study. *Annals of Neurology*.

[18] Longstreth W. T., Arnold A. M., Beauchamp N. J. (2005). Incidence, manifestations, and predictors of worsening white matter on serial cranial magnetic resonance imaging in the elderly. *Stroke*.

[19] Folstein M. F., Folstein S. E., McHugh P. R. (1975). ‘Mini-mental state’: a practical method for grading the cognitive state of patients for the clinician. *Journal of Psychiatric Research*.

[20] Nasreddine Z. S., Phillips N. A., Bédirian V. (2005). The Montreal Cognitive Assessment, MoCA: a brief screening tool for mild cognitive impairment. *Journal of the American Geriatrics Society*.

[21] Nagaratnam J. M., Sharmin S., Diker A., Lim W. K., Maier A. B. (2022). Trajectories of Mini-Mental State Examination scores over the lifespan in general populations: a systematic review and meta-regression analysis. *Clinical Gerontologist*.

[22] Aiello E. N., Pasotti F., Appollonio I., Bolognini N. (2022). Trajectories of MMSE and MoCA scores across the healthy adult lifespan in the Italian population. *Aging Clinical and Experimental Research*.

[23] Aiello E. N., Pasotti F., Appollonio I., Bolognini N. (2022). Equating mini-mental state examination (MMSE) and Montreal cognitive assessment (MoCA) scores: conversion norms from a healthy Italian population sample. *Aging Clinical and Experimental Research*.

[24] Galioto R., Garcia S., Spitznagel M. B. (2014). The Mini-Mental State Exam (MMSE) is not sensitive to cognitive impairment in bariatric surgery candidates. *Surgery for Obesity and Related Diseases*.

[25] Laks J., Coutinho E. S. F., Junger W. (2010). Education does not equally influence all the Mini Mental State Examination subscales and items: inferences from a Brazilian community sample. *Revista Brasileira de Psiquiatria*.

[26] Fan H., Meng Y., Zhu L., Fan M., Wang D., Zhao Y. (2024). A review of methods for assessment of cognitive function in high-altitude hypoxic environments. *Brain and Behavior*.

[27] Davis D. H., Creavin S. T., Yip J. L., Noel-Storr A. H., Brayne C., Cullum S. (2015). Montreal Cognitive Assessment for the diagnosis of Alzheimer’s disease and other dementias. *Cochrane Database of Systematic Reviews*.

[28] Pires L. A., Almeida A. L. M. d., Paraízo M. d. A. (2022). Cross-sectional assessment of mild cognitive impairment in pre-dialysis chronic kidney disease and its association with inflammation and changes seen on MRI: what the eyes cannot see. *Brazilian Journal of Nephrology*.

[29] Pinto T. C. C., Santos M. S. P., Machado L. (2019). Optimal cutoff scores for dementia and mild cognitive impairment in the Brazilian version of the Montreal Cognitive Assessment among the elderly. *Dementia and Geriatric Cognitive Disorders Extra*.

[30] Brucki S. M. D., Nitrini R., Caramelli P., Bertolucci P. H. F., Okamoto I. H. (2003). Sugestões para o uso do mini-exame do estado mental no Brasil. *Arquivos de Neuro-Psiquiatria*.

[31] Crockett R. A., Hsu C. L., Dao E. (2021). Painting by lesions: white matter hyperintensities disrupt functional networks and global cognition. *NeuroImage*.

[32] Li Q.-Q., Wu K., Xu J.-L., Yin L. (2022). White matter damage in patients with mild cognitive impairment in Parkinson’s disease. *Quantitative Imaging in Medicine and Surgery*.

[33] Wang J., Liang Y., Chen H. (2019). Structural changes in white matter lesion patients and their correlation with cognitive impairment. *Neuropsychiatric Disease and Treatment*.

[34] Malta M., Cardoso L. O., Bastos F. I., Magnanini M. M. F., Silva C. M. F. P. d. (2010). Iniciativa STROBE: subsídios para a comunicação de estudos observacionais. *Revista de Saúde Pública*.

[35] Vandenbroucke J. P., von Elm E., Altman D. G. (2014). Strengthening the reporting of observational studies in epidemiology (STROBE): explanation and elaboration. *International Journal of Surgery*.

[36] Vandenbroucke J. P., von Elm E., Altman D. G. (2007). Strengthening the reporting of observational studies in epidemiology (STROBE): explanation and elaboration. *PLoS Medicine*.

[37] von Elm E., Altman D. G., Egger M., Pocock S. J., Gøtzsche P. C., Vandenbroucke J. P. (2008). The Strengthening the Reporting of Observational Studies in Epidemiology (STROBE) statement: guidelines for reporting observational studies. *Journal of Clinical Epidemiology*.

[38] Kalache A., Gatti A. (2003). Active ageing: a policy framework. *Advances in Gerontology Uspekhi Gerontologii*.

[39] Cunha J. A. (2001). *Manual da versão em português das Escalas Beck*.

[40] Hughes C. P., Berg L., Danziger W. L., Coben L. A., Martin R. L. (1982). A new clinical scale for the staging of dementia. *British Journal of Psychiatry*.

[41] Morris J. C. (1993). The Clinical Dementia Rating (CDR): current version and scoring rules. *Neurology*.

[42] Katz S., Ford A. B., Moskowitz R. W., Jackson B. A., Jaffe M. W. (1963). Studies of illness in the aged. The index of ADL: a standardized measure of biological and psychosocial function. *JAMA*.

[43] Lawton M. P., Brody E. M. (1969). Assessment of older people: self-maintaining and instrumental activities of daily living. *The Gerontologist*.

[44] Barroso W. K. S., Rodrigues C. I. S., Bortolotto L. A. (2021). Brazilian guidelines of hypertension-2020. *Arquivos Brasileiros de Cardiologia*.

[45] Benedetti T. R. B., Antunes P. d. C., Rodriguez-Añez C. R., Mazo G. Z., Petroski É. L. (2007). Reprodutibilidade e validade do Questionário Internacional de Atividade Física (IPAQ) em homens idosos. *Revista Brasileira de Medicina do Esporte*.

[46] Tombaugh T. (2004). Trail Making Test A and B: normative data stratified by age and education. *Archives of Clinical Neuropsychology*.

[47] Tombaugh T. N., McIntyre N. J. (1992). The mini-mental state examination: a comprehensive review. *Journal of the American Geriatrics Society*.

[48] Sakurai R., Kim Y., Inagaki H. (2021). MMSE cutoff discriminates hippocampal atrophy: neural evidence for the cutoff of 24 Points. *Journal of the American Geriatrics Society*.

[49] Vyas A., Aisopos F., Vidal M.-E., Garrard P., Paliouras G. (2021). Calibrating Mini-Mental State Examination scores to predict misdiagnosed dementia patients. *Applied Sciences*.

[50] Thomann A. E., Berres M., Goettel N., Steiner L. A., Monsch A. U. (2020). Enhanced diagnostic accuracy for neurocognitive disorders: a revised cut-off approach for the Montreal Cognitive Assessment. *Alzheimer’s Research & Therapy*.

[51] Wahlund L. O., Barkhof F., Fazekas F. (2001). A new rating scale for age-related white matter changes applicable to MRI and CT. *Stroke*.

[52] Koedam E. L. G. E., Lehmann M., Van Der Flier W. M. (2011). Visual assessment of posterior atrophy development of a MRI rating scale. *European Radiology*.

[53] Scheltens P., Launer L. J., Barkhof F., Weinstein H. C., Gool W. A. (1995). Visual assessment of medial temporal lobe atrophy on magnetic resonance imaging: interobserver reliability. *Journal of Neurology*.

[54] Armitage P., Berry G., Matthews J. N. S. (2013). *Statistical Methods in Medical Research*.

[55] Watfa G., Husson N., Buatois S., Laurain M. C., Miget P., Benetos A. (2011). Study of mini-mental state exam evolution in community-dwelling subjects aged over 60 years without dementia. *The Journal of Nutrition, Health & Aging*.

[56] Ranson J. M., Kuźma E., Hamilton W., Muniz-Terrera G., Langa K. M., Llewellyn D. J. (2019). Predictors of dementia misclassification when using brief cognitive assessments. *Neurology Clinical Practice*.

[57] Pinto T. C. C., Machado L., Bulgacov T. M. (2019). Is the Montreal cognitive assessment (MoCA) screening superior to the mini-mental state examination (MMSE) in the detection of mild cognitive impairment (MCI) and alzheimer’s disease (AD) in the elderly?. *International Psychogeriatrics*.

[58] Julayanont P., Tangwongchai S., Hemrungrojn S. (2015). The Montreal Cognitive Assessment-Basic: a screening tool for mild cognitive impairment in illiterate and low-educated elderly adults. *Journal of the American Geriatrics Society*.

[59] Malek-Ahmadi M., Nikkhahmanesh N. (2024). Meta-analysis of Montreal Cognitive Assessment diagnostic accuracy in amnestic mild cognitive impairment. *Frontiers in Psychology*.

[60] Yeung P. Y., Wong L. L., Chan C. C. (2020). Montreal Cognitive Assessment-single cutoff achieves screening purpose. *Neuropsychiatric Disease and Treatment*.

[61] Jia X., Wang Z., Huang F. (2021). A comparison of the Mini-Mental State Examination (MMSE) with the Montreal Cognitive Assessment (MoCA) for mild cognitive impairment screening in Chinese middle-aged and older population: a cross-sectional study. *BMC Psychiatry*.

[62] Breton A., Casey D., Arnaoutoglou N. A. (2019). Cognitive tests for the detection of mild cognitive impairment (MCI), the prodromal stage of dementia: meta-analysis of diagnostic accuracy studies. *International Journal of Geriatric Psychiatry*.

[63] Roh H. W., Choi J. g., Kim N. R. (2020). Associations of rest-activity patterns with amyloid burden, medial temporal lobe atrophy, and cognitive impairment. *EBioMedicine*.

[64] Zhang L., Sun W. H., Xing M. (2019). Medial temporal lobe atrophy is related to learning strategy changes in amnestic mild cognitive impairment. *Journal of the International Neuropsychological Society*.

[65] Rao Y. L., Ganaraja B., Murlimanju B. V., Joy T., Krishnamurthy A., Agrawal A. (2022). Hippocampus and its involvement in Alzheimer’s disease: a review. *3 Biotech*.

[66] Chauveau L., Kuhn E., Palix C. (2021). Medial temporal lobe subregional atrophy in aging and Alzheimer’s disease: a longitudinal study. *Frontiers in Aging Neuroscience*.

[67] Squire L. R., Stark C. E. L., Clark R. E. (2004). The medial temporal lobe. *Annual Review of Neuroscience*.

[68] Chaudhary S., Zhornitsky S., Chao H. H., van Dyck C. H., Li C.-S. R. (2022). Emotion processing dysfunction in Alzheimer’s disease: an overview of behavioral findings, systems neural correlates, and underlying neural biology. *American Journal of Alzheimer’s Disease and Other Dementias*.

[69] Chorghay Z., Káradóttir R. T., Ruthazer E. S. (2018). White matter plasticity keeps the brain in tune: axons conduct while glia wrap. *Frontiers in Cellular Neuroscience*.

[70] Arai K., Hamanaka G., Ohtomo R., Takase H., Lok J. (2018). White-matter repair: interaction between oligodendrocytes and the neurovascular unit. *Brain Circulation*.

[71] Pansieri J., Hadley G., Lockhart A., Pisa M., DeLuca G. C. (2023). Regional contribution of vascular dysfunction in white matter dementia: clinical and neuropathological insights. *Frontiers in Neurology*.

[72] Kynast J., Lampe L., Luck T. (2018). White matter hyperintensities associated with small vessel disease impair social cognition beside attention and memory. *Journal of Cerebral Blood Flow and Metabolism*.

[73] Platero C., Tobar M. C. (2020). Longitudinal survival analysis and two-group comparison for predicting the progression of mild cognitive impairment to Alzheimer’s disease. *Journal of Neuroscience Methods*.

[74] Strand B. H., Knapskog A. B., Persson K. (2018). Survival and years of life lost in various aetiologies of dementia, mild cognitive impairment (MCI) and subjective cognitive decline (SCD) in Norway. *PLoS One*.

[75] Hshieh T. T., Jung W. F., Grande L. J. (2018). Prevalence of cognitive impairment and association with survival among older patients with hematologic cancers. *JAMA Oncology*.

[76] Gillis C., Mirzaei F., Potashman M., Ikram M. A., Maserejian N. (2019). The incidence of mild cognitive impairment: a systematic review and data synthesis. *Alzheimer’s and Dementia: Diagnosis, Assessment & Disease Monitoring*.

[77] Klainin-Yobas P., Kowitlawakul Y., Lopez V. (2019). The effects of mindfulness and health education programs on the emotional state and cognitive function of elderly individuals with mild cognitive impairment: a randomized controlled trial. *Journal of Clinical Neuroscience*.

[78] Matyas N., Keser Aschenberger F., Wagner G. (2019). Continuing education for the prevention of mild cognitive impairment and Alzheimer’s-type dementia: a systematic review and overview of systematic reviews. *BMJ Open*.

[79] Luz A. L. d. A., Silva-Costa A., Barbosa E. L., Marques L. P., Souto E. P., Griep R. H. (2022). Função cognitiva e controle da pressão arterial em idosos hipertensos. *Ciência & Saúde Coletiva*.

[80] Hartle L., Charchat-Fichman H. (2021). Mild cognitive impairment history and current procedures in low- and middle-income countries: a brief review. *Dementia & Neuropsychologia*.

[81] Bowie C. R., Harvey P. D. (2006). Administration and interpretation of the Trail making test. *Nature Protocols*.

[82] MacPherson S. E., Allerhand M., Cox S. R., Deary I. J. (2019). Individual differences in cognitive processes underlying Trail Making Test-B performance in old age: the Lothian Birth Cohort 1936. *Intelligence*.

[83] Bednorz A., Religa D. (2023). Utility of the comprehensive Trail Making Test in the assessment of mild cognitive impairment in older patients. *Geriatrics*.

[84] Kabiri S., Jameie M., Balali P. (2023). Trail Making Test could predict impairment in cognitive domains in patients with multiple sclerosis: a study of diagnostic accuracy. *Archives of Clinical Neuropsychology*.

[85] Hafiz N. J., Lohse A., Haas R. (2023). Trail Making Test error analysis in subjective cognitive decline, mild cognitive impairment, and Alzheimer’s dementia with and without depression. *Archives of Clinical Neuropsychology*.

